# Immunomodulatory effects of 4-hydroxynonenal

**DOI:** 10.1016/j.redox.2025.103719

**Published:** 2025-06-06

**Authors:** Melina Ioannidis, Johanna Tjepkema, Michael R.P. Uitbeijerse, Geert van den Bogaart

**Affiliations:** a Department of Molecular Immunology, Groningen Biomolecular Science and Biotechnology Institute, University of Groningen, Nijenborgh 7, 9747AG, Groningen, the Netherlands; b Department of Medical Biology and Pathology, University Medical Centre Groningen, Groningen, the Netherlands

**Keywords:** 4-Hydroxy-2-nonenal, 4-HNE, Immune signaling, Infection, ROS

## Abstract

The reactive aldehyde 4-hydroxy-2-nonenal (4-HNE) is a byproduct of lipid peroxidation driven by reactive oxygen species (ROS). 4-HNE covalently binds to macromolecules such as proteins, altering their functions. While 4-HNE is implicated in various ROS-related pathologies, its impact on the immune system remains incompletely understood. This review explores how 4-HNE influences molecular mechanisms involved in inflammation and immune cell functions. 4-HNE modulates inflammation through the interaction with several signaling pathways, including nuclear factor kappa-light-chain enhancement of activated B cells (NF-κB), nuclear factor erythroid 2-related factor (Nrf2), mitogen-activated protein kinases (MAPK), toll-like receptor (TLR) 4, and stimulator of interferon genes (STING), thereby affecting immune responses and modulating cytokine production and inflammasome activation. However, its effects are complex, exhibiting both pro- and anti-inflammatory properties depending on dose and cell type. This review highlights the multiple mechanisms by which 4-HNE modulates the immune cells' responses.

## Highlights

• 4-HNE modulates multiple immune signaling pathways including TLR4, NF-κB, Nrf2, MAPKs, STING and inflammasome.
• Pathology and immune modulation by 4-HNE are specific for the immune cell type.
• Dose- and cell type-dependent research is essential to determine the therapeutic potential of 4-HNE in immune-related diseases.

## 1 Introduction

The immune system protects our body against pathogens or tissue injury by recognizing pathogenic structures through pathogen-associated molecular patterns (PAMPS) and cell damage via damage-associated molecular patterns (DAMPs). The first line of defense that recognizes PAMPs and DAMPs is the innate (natural or native) immune system. The innate immune system includes dendritic cells, neutrophils, eosinophils, basophils, mast cells, natural killer cells, and macrophages [1]. The adaptive immune system, or acquired immune system, is a highly specific defense mechanism that includes B and T lymphocytes that recognize specific antigens. Recognition of the antigen stimulates the production of antibodies by B lymphocytes. Via the stimulation of apoptosis by granzyme B and perforin or Fas receptor interactions with FasL in the target cells, cytotoxic T lymphocytes eliminate infected, damaged, or cancerous cells. Helper T cells stimulate the production of antibodies and cytokines that neutralize the pathogen and also stimulate other immune cells [2].

The immune response is tightly regulated via distinct signaling pathways. Upon PAMP (e.g., the bacterial glycolipid lipopolysaccharide (LPS)) or DAMP (e.g., HMGB1, S100 proteins) recognition, intracellular signaling cascades are activated through myeloid differentiation primary response 88 (MyD88)- and TIR-domain-containing adapter inducing interferon (TRIF)-dependent pathways, leading to the activation of NF-κB, MAPK, and interferon regulatory factors (IRFs). These transcription factors drive the expression of pro-inflammatory cytokines (e.g., tumor necrosis factor (TNF)-α, Interleukin (IL)-6, IL-1β) and type I interferons (e.g., interferon (IFN)- α and -β) while also inducing reactive oxygen species (ROS) production. ROS generation occurs via NADPH oxidase (NOX) activation and mitochondrial metabolic reprogramming, further amplifying inflammatory responses and antimicrobial activity. However, excessive ROS production can also contribute to oxidative stress and immune dysregulation, reinforcing the importance of tightly regulated signaling mechanisms [[3], [4], [5]].

**Fig. 1:**
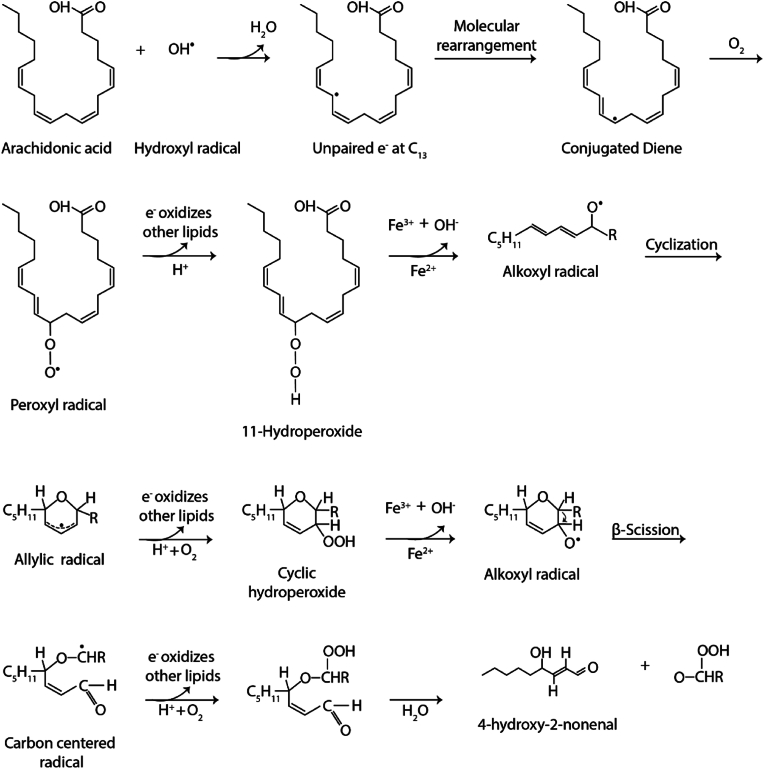
**Potential mechanism for the formation of 4-HNE from arachidonic acid.** Initially, a hydroxyl radical (•OH) can abstract a hydrogen atom from arachidonic acid, creating a carbon-centered radical at carbon 13 (C13). This radical undergoes molecular rearrangement, forming a conjugated diene, which can react with molecular oxygen (O_2_) to yield a peroxyl radical. The hydrogenation of this peroxyl radical generates 11-hydroperoxide, which, in the presence of iron, is converted into an alkoxyl radical. This intermediate undergoes cyclization, resulting in the formation of an allylic radical. Upon further exposure to oxygen and hydrogen, the allylic radical is transformed into another hydroperoxide, which again reacts with iron to form a second alkoxyl radical. This alkoxyl radical undergoes β-scission, leading to the formation of a new carbon-centered radical. Further peroxidation of this radical yields an unstable intermediate, which, upon hydrolysis, results in the formation of 4-hydroxynonenal (4-HNE). Besides 4-HNE, other products can be formed during the final hydrolysis step, which are likely unstable and may further degrade into additional compounds.

ROS are essential signaling molecules. Three main types of ROS are superoxide anion (O_2_^•^^-^), hydrogen peroxide (H_2_O_2_), and hydroxyl radicals (OH^•^^-^) [6]. Physiological production of ROS is essential to maintain cellular processes like cell signaling, differentiation, proliferation, apoptosis, and cytoskeleton regulation [7]. Immune cells utilize increased ROS production to eliminate pathogens through the so-called oxidative burst [8,9]. However, excessive ROS levels can damage cellular components like proteins, lipids, and nucleic acids [10]. Due to their high reactivity, ROS can react with different biomolecules. One of the biomolecules that ROS react with are polyunsaturated fatty acids of cellular membranes, like linoleic or arachidonic acid. The oxidation of omega-6 polyunsaturated lipids by ROS can produce the reactive aldehyde 4-HNE (Fig. 1). Under normal physiological conditions, the concentration of 4-HNE is between 0.05 and 15 μM in human blood and serum [11]. However, during oxidative stress, the concentration of 4-HNE can rise to more than 100 μM [12,13].

**Fig. 2:**
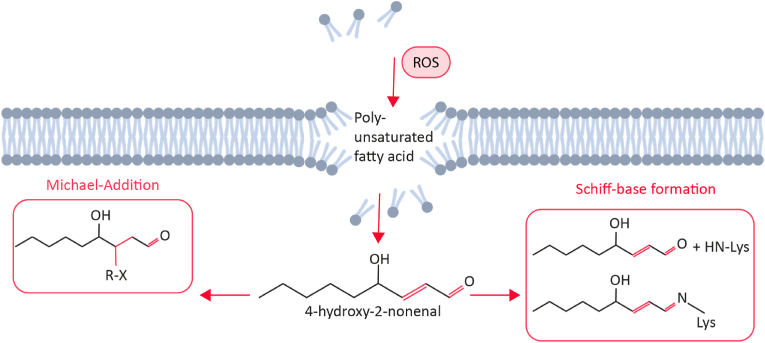
**4-HNE is a reactive aldehyde.** ROS produces 4-HNE, a reactive aldehyde, via the oxidation of polyunsaturated fatty acids. Once 4-HNE is formed, protein adducts can be formed by Michaelis-Menten addition or Schiff base formation. Image was created with BioRender.com.

**Fig. 3:**
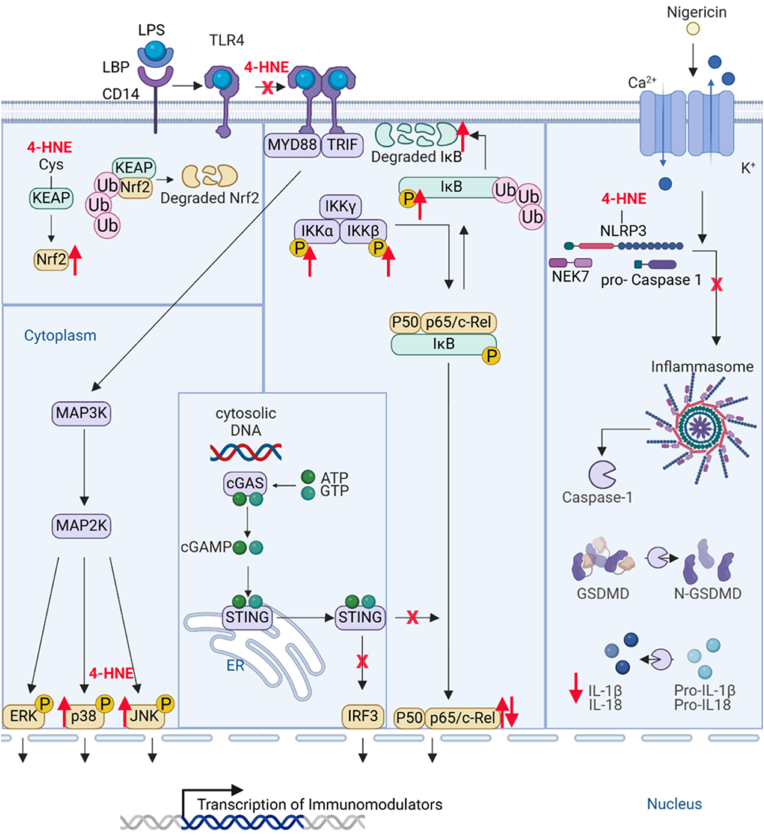
**4-HNE affects key signaling pathways influencing the transcription of several immunomodulators.** This figure summarizes the immunomodulatory effects of 4-HNE on key signaling pathways. 4-HNE inhibits TLR dimerization, blocking downstream immune activation. In the NF-κB pathway, low doses promote activation via IκB degradation, while high doses inhibit NF-κB. 4-HNE activates Nrf2 by binding KEAP1, promoting antioxidant enzyme expression (HO-1, SOD, GST). In MAPK signaling, 4-HNE increases p38 phosphorylation, activates JNK in specific cells, and inhibits ERK in immune cells while activating it in others. Also, 4-HNE blocks NLRP3 inflammasome activation by preventing NEK7 interaction and inhibits STING signaling. Image was created with BioRender.com.

4-HNE is a reactive α- β-aldehyde due to three reactive groups: a carbonyl group at C1, a hydroxyl group at C4, and a double bond between C2 and C3 (Fig. 2). These functional groups make 4-HNE electrophilic and highly reactive towards nucleophilic thiol and amino groups. Therefore, 4-HNE can interact with other molecules through two distinct mechanisms: the slow and reversible formation of a Schiff base or Michael addition, in which thiol or amino compounds react at the C3 position of the double bond (Fig. 2). Hence, 4-HNE can form adducts with proteins containing histidine, lysine, and cysteine residues, lipids containing amino groups, and nucleic acids, predominantly via guanosines [14]. Detoxification of 4-HNE by the glutathione (GSH) and glutathione-S-transferase (GST) system is important in preventing protein interactions with 4-HNE. 4-HNE spontaneously reacts with GSH and forms a conjugate, thereby preventing 4-HNE from interacting with other molecules. However, this reaction is more rapid in the presence of the GST enzyme [15]. This regulation system for 4-HNE is essential to prevent oxidative damage in cells (see Fig. 3).

Since 4-HNE can form adducts with proteins, it can alter their functions, and depending on the concentration and the cell type (e.g., immune cells, epithelial cells, or fibroblasts), 4-HNE modulates distinct cellular pathways. This is well recognized to play a role in disease, and elevated levels of 4-HNE adducts have been found in ROS-related pathologies, including Alzheimer's disease [16], atherosclerosis [17], and sepsis [18]. While 4-HNE itself may not be the primary initiator of inflammation in these pathologies, its role in affecting the direction of inflammatory response has become apparent over the years. Originating as a by-product of inflammation-related ROS production, 4-HNE can modulate cellular signaling pathways involved in inflammation, such as TLRs, NF-κB, and MAPKs. This review will discuss the importance of 4-HNE in regulating the inflammatory response, with a focus on its effects in macrophages, monocytes, and other immune cells.

## 2 4-HNE interference with Toll-like receptor signaling

Immune cells recognize PAMPs and DAMPs due to the presence of so-called pattern recognition receptors (PRR). TLRs are a class of PRRs that initiate the immune response by triggering diverse signaling pathways [19]. For instance, macrophages can recognize LPS via TLR4. Since LPS is a glycolipid, it naturally aggregates in aqueous environments, making it less accessible for TLR4. Therefore, LPS-binding protein (LBP) and the membrane receptor CD14 extract LPS from aggregates and monomerize it to make it accessible for TLR4 for effective signaling [[20], [21], [22]]. Once TLR4 binds to LPS, the dimerization of two TLR4 molecules triggers intracellular signaling, including MYD88-and TRIF-dependent cascades that activate NF-κB, MAPK, and IRF. These transcription factors provoke the production of pro-inflammatory cytokines and type I interferons and promote the generation of ROS [23,24].

Upon TLR4 signaling, NOX enzymes are also activated to produce cytoplasmic ROS, leading to the generation of superoxide, which can be converted into hydrogen peroxide. At the same time, redirection of the electron transport chain in mitochondria leads to increased ROS production via Complexes I and III. The increased ROS production results in elevated levels of lipid peroxidation products like 4-HNE [25,26], suggesting a role for 4-HNE in regulating TLR signaling. Indeed, co-immunoprecipitation of GFP-tagged TLR4 and FLAG-tagged TLR4 in the Ba/F3 murine hybridoma cell line revealed that 10 μM and 20 μM of 4-HNE decrease dimerization of TLR4. To assess the effect on downstream TLR4 signaling, RAW264.7 murine macrophages were transfected with the reporter construct containing a binding site for NF-κB or IRF3 and pre-treated with 4-HNE (1 μM, 5 μM, 10 μM, 20 μM) for 1 h before treatment with LPS (10 ng/mL). The assay revealed a dose-dependent downregulation of NF-κB and IRF3 signaling when treated with 4-HNE, with the highest effect observed at 20 μM 4-HNE, indicating that TLR4 signaling is downregulated by 4-HNE [26].

However, the opposite has also been reported, and 4-HNE can also increase TLR signaling. Intraperitoneal injection of mice with dextran sodium sulfate (DSS)-induced colitis, a model of inflammatory bowel disease (IBD), with 4-HNE (32 μmol/kg/day) resulted in an increased severity of the colitis. The mice showed a reduced colon length, increased pro-inflammatory cytokine levels like IL-6, and increased infiltration of CD45^+^ F4/80^+^ monocytes in the colon. HEK-Blue mTLR4 cells, a TLR4 reporter cell line that secretes alkaline phosphatase after TLR4 activation via the NF-κB signaling pathway [27], were treated with plasma of the 4-HNE-treated murine colitis model. Compared to plasma from mice not injected with 4-HNE, plasma from 4-HNE-treated DSS mice showed increased TLR-4 activation [28]. Another study showed using real-time RT-PCR that treatment of the human monocyte cell line U937 with 5 μM 4-HNE for 4 h and 6 h significantly increased mRNA levels of TLR4. In addition, 4-HNE treatment induced TLR4 activation, measured through cytokine production after 24 h of treatment. These effects on cytokine production could be decreased with an anti-TLR4 antibody or siRNA against TLR4 [29]. In cortical neurons, the mRNA and protein expression of TLR2 and TLR4 were also increased by 4-HNE (5–10 μM), where 4-HNE resulted in elevated levels of TLR2 and TLR4 after 3 h of treatment and remained elevated through 20 h of treatment [30].

These studies imply that 4-HNE has a role in TLR4 signaling but report opposite effects. These differences might be due to handling differences, because 4-HNE is highly reactive and can form adducts with proteins present in the grown medium or circulation, and some effects might therefore be indirect [31,32]. Moreover, 4-HNE might influence TLR4 in a dose-dependent and cell type-dependent manner, as different 4-HNE detoxification rates are reported for different cells [33,34]. However, further research is needed to explore the effect of 4-HNE on TLR4 and other TLRs. Specifically, it remains to be determined to what extent 4-HNE directly modifies TLRs, forms oxidation-specific epitopes that could serve as novel TLR ligands [35], or impacts downstream signaling pathways. These potential mechanisms should be examined better to understand the role of oxidative damage in immune signaling.

## 3 4-HNE and its role in NF-κB signaling

The transcription factor NF-κB influences innate and adaptive immune functions by regulating the expression of pro-inflammatory genes, including genes encoding cytokines, chemokines, and inflammasome regulatory factors. Other processes where NF-κB plays important roles in innate immune cells include survival, activation, and differentiation. Consequently, dysregulating or modulating the NF-κB signaling pathway strongly affects immune cells [36].

The NF-κB signaling pathway can be evoked by several signaling pathways, including TLRs [37], and is distinguished into non-canonical (alternative) and canonical (classical) signaling pathways [38]. The classical pathway depends on the IκB kinase adaptor molecule Nf-κB essential modulator (NEMO/IKK-γ), whereas the alternative pathway is independent of NEMO [39]. The canonical NF-κB pathway is activated by stimulating receptors like TLR. Receptor activation leads to the recruitment and phosphorylation of the IKK complex, consisting of NEMO, IKKα, and IKKβ [40,41]. The activated IKK complex then phosphorylates IκB, a family of NF-κB inhibitory proteins (e.g of IκB-α, -β, and -ε). In the case of IκB-α, phosphorylation occurs at specific amino-terminal serine residues Ser32 and Ser36. The phosphorylated IκBα is then ubiquitinated at lysines 21 and 22, which target IκB for degradation by the 26S proteasome. The degradation of IκB releases the NF-κB dimer, allowing it to translocate to the nucleus, where it stimulates gene expression [40,41].

By contrast, the non-canonical NF-κB signaling pathway is only triggered by certain receptors, like receptor activator of NF-κB (RANK). Upon activation of this receptor, the TNF receptor-associated factor (TRAF)2 and TRAF3 complexes are ubiquitinated and degraded by the proteasome. The degradation of TRAF2 and TRAF3 leads to stabilization of NF-κB-inducing kinase (NIK), which in turn phosphorylates IKKα, resulting in the phosphorylation of p100. P100 phosphorylation results in partial degradation of p100 and the generation of p52. P52 can form a dimer with Rel/B and translocate to the nucleus, activating gene expression [42].

Depending on the concentration and cell type, 4-HNE differentially affects the NF-κB signaling pathway and influences inflammatory responses. For instance, western blotting revealed that treatment of the murine macrophage cell line RAW264.7 with 1 μM of 4-HNE resulted in the phosphorylation of IKK-α/β. This phosphorylation of IKK-α/β allows NF-κB transfer into the nucleus and hence increases NF-κB activity. Indeed, an increase in NF-κB activity was measured after 4-HNE treatment in RAW264.7 cells transfected with the NF-κB SEAP reporter gene. The NF-κB SEAP reporter gene can be used to measure NF-κB activity by detecting the secretion of alkaline phosphatase (SEAP) into the supernatant, which is proportional to the activation of the NF-κB pathway and the transcription of the SEAP gene driven by NF-κB-responsive promoters [43].

In contrast, in the human monocytic leukemia cell line (THP-1) and freshly isolated human monocytes, treatment with 4-HNE followed by 1 h incubation with the TLR-4 ligand LPS resulted in a dose-dependent inhibition of NF-κB, measured by an electrophoretic mobility shift assay, which measures NF-κB binding to nucleic acids. LPS-induced NF-κB activity was reduced slightly when monocytes were pre-treated with 6.25 μM 4-HNE, whereas 12.5 μM and 25 μM significantly decreased, and 50 μM 4-HNE completely terminated LPS-induced NF-κB activation [44].

Interestingly, the inhibition of NF-κB by 4-HNE was dependent on the stimuli. THP-1 cells were pre-treated with 25 μM 4-HNE, followed by treatment with LPS, IL-1β, Phorbol 12-myristate 13-acetate (PMA), or TNF-α. Pre-treatment with 4-HNE resulted in a decreased activation of NF-κB activity when treated with LPS, IL-1β, and PMA (4 h and 12 h), but treatment with TNF-α showed a 20 % increase in NF-κB signaling. In addition to NF-κB activation, the phosphorylation state of IκB-α, -β, and -ε were determined by Western blot analysis. The stimulation of THP-1 with LPS for 1 h resulted in proteolysis of IκB-α, -β, and -ε, which was prevented by pre-treatment with 25 μM 4-HNE. However, 4-HNE did not stimulate IκB-α phosphorylation, which was seen when THP-1 cells were treated with proteasome inhibitor PSI (Z-Ile-Glu(OtBu)-Ala-Leu-al). Moreover, treatment with TNF-α did not reduce the proteolysis of IκB-α in cells pre-treated with 4-HNE [44].

In other cell types, 4-HNE also reduced NF-κB activity. Primary Kupffer cells, liver resident macrophages, were isolated from rat liver and pre-treated for 1 h with 4-HNE (0.1–10 μM) before the cells were treated for 1 h with LPS. The treatment of 4-HNE resulted in a dose-dependent decrease of NF-κB activity compared to LPS alone, where treatment with 5 μM resulted in a 20 % reduction. Moreover, Western blot revealed that the treatment with 4-HNE resulted in the stabilization of IκB-α and a reduction in phosphorylation of IκB-α [45] as seen previously in THP-1 cells [44]. The immunoprecipitation of p65 with IκBα supported these results, showing that a high concentration of 4-HNE (10 μM) resulted in less interaction of p65 and IκBα [45].

In the human lymphoma Jurkat T cell line, pre-treatment with 30 μM of 4-HNE also lowered activation of the NF-κB pathway by the PKC activator tetradecanoyl phorbol acetate and the ionophore ionomycin, as apparent from higher IκB-α protein levels and decreased phosphorylation [46].

Overall, these results suggest that 4-HNE impacts NF-κB in a concentration-dependent manner. At low concentrations, 4-HNE increases NF-κB activity in RAW264.7 cells by forming adducts with the IKK, promoting IκB degradation. However, at high concentrations, 4-HNE decreases NF-κB activity in immune cells. However, this model is challenged by controversial reports showing an increased NF-κB activity in rat prostate endothelial cells treated with high concentrations of 4-HNE (15 μM) [47], suggesting that 4-HNE not only dose-dependently but also cell-type dependently regulates the NF-κB signaling pathway. The effect of 4-HNE treatment on other NF-κB modulators was not investigated. Given that both the conventional (canonical) and unconventional (non-canonical) NF-κB pathways play distinct roles in immune responses and disease development [39,42], it would be interesting to explore how 4-HNE influences additional regulators within these signaling pathways.

## 4 4-HNE influences Nrf2 signaling via KEAP1

Nuclear factor erythroid 2-related factor (Nrf) 2 is a transcription factor that plays a central role in the cellular defense mechanism against oxidative stress by regulating the expression of antioxidant and detoxification genes. Nrf2 plays an important role in the cellular defense against oxidative stress and modulates the inflammatory response through the stimulation of antioxidant enzymes (HO-1, SOD), and detoxifying enzymes (GST) [48]. Under normal conditions, Nrf2 is bound to Kelch-like-ECH-associated protein (KEAP)1 and is located in the cytoplasm. The binding of KEAP1 to Nrf2 results in continuous ubiquitination by the E3 ubiquitin ligase complex, leading to the proteasomal degradation of Nrf2. As a result, Nrf2 levels stay low and prevent unnecessary activation of the antioxidant system [49].

Upon oxidative stress or electrophilic attack, via Michaelis addition, KEAP1's cysteine residues undergo conformational changes that trigger its dissociation from Nrf2, thereby preventing KEAP1-mediated degradation of Nrf2 and allowing its activation [50]. As a result, Nrf2 accumulates in the cytoplasm and translocates to the nucleus, where it forms a heterodimer with small Maf (sMaf) proteins [49,51], allowing the binding to the antioxidant response element in the promoter regions of target genes. These target genes include the antioxidant enzymes heme oxygenase (HO)-1 and superoxide dismutase (SOD). Nrf2 also stimulates the expression of the glutathione synthesis gene glutamate-cysteine ligase (GCL), which detoxifies 4-HNE [52,53].

There is not much evidence in immune cells on the influence of 4-HNE on the Nrf2 signaling pathways. There are reports that 4-HNE promotes Nrf2 signaling in macrophages and other cell types by suppressing KEAP1. KEAP1 has three distinct cysteines reported to be directly modified by 4-HNE [54]. This is in line with other literature showing the activation of Nrf2 by 4-HNE in different cell types, including HEK-293, HUVEC (human umbilical vein endothelial) cells, and PC12 neuroendocrine cells [50,54]. Supporting this activation of Nrf2 by 4-HNE, in peritoneal macrophages isolated from wild-type mice it was shown that treatment with 20 μM 4-HNE increases the RNA expression of the genes coding for HO-1 and peroxiredoxin (Prx) I, well-known target genes of Nrf2. In contrast, in *nrf2* (−/−) knock-out mice, 4-HNE could not induce expression for HO-1 and Prx I [55]. Moreover, the treatment of alveolar macrophages isolated from C57BL/6J and C3H/HeJ mice showed that concentrations of 25 μM and 50 μM 4-HNE also increased HO-1 protein levels compared to the untreated control [56]. Finally, Nrf2 can suppress inflammation by indirectly reducing protein levels of inducible nitric oxide synthase (iNOS). iNOS is increased in RAW264.7 macrophages stimulated with LPS for 6 h. However, in the presence of 50 μM 4-HNE, LPS did not trigger this iNOS increase, supporting Nrf2 stimulation [57]. Thus, 4-HNE seems to increase NRF2 signaling in macrophages and other cell types.

Together, these findings show that 4-HNE promotes Nrf2 activation across various cell types, including macrophages, and enhances the expression of Nrf2 target genes, ultimately contributing to an anti-inflammatory response.

## 5 MAPK signaling is influenced by 4-HNE

The MAPK signaling pathway is an important signaling cascade involved in the immune response, regulating cytokine production and apoptosis. The three major MAPKs involved in inflammation are the extracellular signal-regulated kinase (ERK), p38 MAPK, and c-Jun N-terminal kinase (JNK) pathways. An extracellular stimulus, like the activation of TLR4 by LPS, triggers these pathways. Upon receptor activation, MAPK kinases kinases (MAP3K) (TAK1, RAF, ASK1) phosphorylate and activate MAPK kinases, including ERK1/2, p38 MAPK, and JNK. Activated MAPK kinases, in turn, activate transcription factors like NF-κB, activator protein (AP)-1, and cAMP response element-binding protein (CREB). These transcription factors drive the production of cytokines, chemokines, adhesion molecules, and other immune modulators [58,59]. 4-HNE has been reported to modify all three major MAPKs.

### 5.1 p38 MAPK

The p38 MAPK pathway plays a crucial role in the immune response by producing pro-inflammatory cytokines upon activation [60]. The activation of p38 MAPK is mediated by several inflammatory stimuli like chemokines, cytokines, and LPS [59].

In the murine macrophage cell line J774A.1, Western blot analysis showed that treatment with 10 μM 4-HNE resulted in the phosphorylation (i.e. a hallmark of activation) of p38 (Thr180/Tyr182) within 5 min. The phosphorylation increased over time and lasted up to 45 min after stimulation. The treatment of 4-HNE did not affect total p38 expression [61]. Consistent with this, another group showed that treatment with 10 μM 4-HNE resulted in the phosphorylation of p38 in the J774A.1 cell line and mouse peritoneal macrophages isolated from C57BL/6J mice [62]. Treatment of the human monocytic cell line THP-1 with 20–40 μM 4-HNE in the presence of LPS also promoted p38 phosphorylation, which was reduced in the presence of p38 MAPK inhibitor SB203580 but stayed unchanged when treated with JNK inhibitor SP600125 or ERK inhibitor PD98059. This p38 phosphorylation was dependent on the presence of reactive 4-HNE, as it could be reduced with antioxidants like N-acetyl-L-cystein (NAC) or N-mercaptopropionylglycine (MPG) [63].

In the murine macrophage cell line RAW264.7, the stimulation with 50 μM 4-HNE also resulted in the increased phosphorylation of p38 (Thr-180/Tyr-182) after 15 min incubation, with the highest increase at 30 min incubation and a downregulation of p38 phosphorylation after 60 min. The phosphorylation was reduced when cells were treated with non-selective kinase inhibitors PP2 and the selective EGFR tyrosine kinase inhibitor AG1478 [57]. The decreased phosphorylation observed with PP2 and AG1478 indicates that both EGFR and other kinases modulated by PP2 may play a role in 4-HNE-induced phosphorylation of p38. This suggests that 4-HNE likely interacts with multiple kinases, including EGFR, which subsequently leads to the activation of p38 as part of a broader signaling cascade.

However, despite that 4-HNE clearly increases p38 phosphorylation, the kinase activity might actually decrease. In a radioactive assay to measure p38 activity, the pulled-down p38 kinase was incubated with a substrate and γ-32P-labeled ATP. Upon activation, the p38 kinase transfers the radioactive phosphate group from γ-32ATP to the substrate, allowing for the detection of kinase activity by measuring the incorporation of the radioactive label. Treatment of MonoMac 6 and peripheral blood monocytes with 10 μM 4-HNE for 1 h before 15 min of LPS stimulation resulted in significant inhibition of p38 activity compared to LPS alone. However, no significant difference was seen in the phosphorylation of p38 in those cells [64].

Thus, 4-HNE consistently seems to enhance p38 phosphorylation in murine and human monocytes and macrophages, and this can be reversed by antioxidants or specific p38 MAPK inhibitors. However, the γ^32^^P^ATP kinase activity assay, which is a more direct measure of kinase function, showed a decrease in p38 activity upon 4-HNE treatment. This discrepancy suggests that while phosphorylation levels indicate activation, additional regulatory mechanisms influence the functional outcome. Therefore, further investigation across different cell types is necessary to elucidate the impact of 4-HNE on p38 MAPK activity.

### 5.2 JNK

JNK was initially called stress-activated protein kinase (SAPK) since it is activated in response to stress [65]. Later, it was discovered that JNK responds to cytokines, such as TNF-α, IL-1β, and transforming growth factor (TGF)-β. These signals can activate MAP3Ks, like transforming growth factor-β activated kinase (TAK) 1, which phosphorylates and activates MAP2K (MKK4, MKK7), ultimately resulting in the activation of JNK [66]. The activation of JNK triggers the production of pro-inflammatory cytokines, including IL-2, IL-6, and TNF-α, as well as the maturation and activation of T cells. It also mediates inflammasome activation through phosphorylating NLRP3 [67].

Determination of JNK activity using the γ^32^^P^ATP kinase activity assay showed that 10 μM 4-HNE induces JNK activity in the presence and absence of LPS in MonoMac6 cells, without significant changes of phosphorylation of two different isoforms of JNK (p46 and p54) [64]. In the human T lymphoblast cell line CRL2571, treatment of 20 μM 4-HNE increased JNK phosphorylation (Ser63) after 30 min, which was maintained up to 120 min after treatment. Treatment of those cells with different concentrations of 4-HNE showed that 5 μM of 4-HNE is already enough to provoke JNK phosphorylation (Ser63). When treating those cells with the JNK inhibitor SP600125, the effect of 4-HNE-induced apoptosis was inhibited, supporting the activation of JNK by 4-HNE in CRL2571 cells [68].

Besides these few studies on how 4-HNE regulates JNK in immune cells, multiple studies investigated the role of 4-HNE in JNK signaling in other cell types. In the rat neuroendocrine cell line PC12, 25 μM of 4-HNE triggered phosphorylation of JNK measured by Western blot [69]. Moreover, treatment with 10 μM of 4-HNE in human hepatic stellate cells resulted in nuclear translocation of JNK and increased JNK activity [70]. Thus, 4-HNE can increase JNK activity in both immune cells and other cell types. However, the mechanism of how JNK is regulated in immune cells should be investigated further. In human hepatic stellate cells, pull-down of JNK revealed the formation of 4-HNE-histidine adducts, indicating that 4-HNE directly forms protein adducts with JNK and thereby potentially directly regulates JNK [70].

### 5.3 ERK

Like the other MAPKs, ERK regulates cell proliferation, differentiation, survival, and apoptosis. The ERK signaling pathway is regulated by Ras and RAF, which phosphorylate MEK1/2, leading to the phosphorylation of ERK1/2 at Tyr204/187 and Thr202/185, with both phosphorylation sites being necessary for full activation of ERK. ERK1 and ERK2 are promiscuous and can phosphorylate dozens of cytoplasmic and nuclear substrates, including regulatory molecules and transcription factors [71]. As ERK regulates multiple transcription factors, it can also influence the inflammatory response. For instance, it has been shown that ERK can target and activate NF-κB, while its effects on JNK activity vary, either enhancing or inhibiting JNK signaling depending on the cellular context and specific pathways involved [[72], [73], [74], [75]].

Little is known of whether 4-HNE regulates ERK activity in immune cells. However, several studies showed that 4-HNE stimulates ERK signaling in hepatocytes [76], pulmonary [77], and intestinal epithelial cells [78]. In contrast to these observations, treatment of the human monocytic cell line MonoMac6 with 10 μM 4-HNE significantly inhibited LPS-induced ERK activity, measured by the radioactive kinase activity assay. However, treatment with 4-HNE did not affect the phosphorylation of ERK [64].

Thus, at least in MonoMac6 monocytes, 4-HNE seems to inhibit ERK signaling, whereas in other cell types, ERK is stimulated by 4-HNE. These results might suggest that 4-HNE influences ERK in a strongly cell type-dependent fashion. As ERK modulates the immune response, it is important to investigate the precise roles of 4-HNE in ERK signaling of different immune cells.

## 6 Modulation of the immune reaction by 4-HNE

Given that 4-HNE affects NF-κB signaling and the MAPKs, it is not surprising that it modulates the inflammatory response in immune cells.

### 6.1 Inflammasome

The inflammasome is a multiprotein complex responsible for a pro-inflammatory response and cell death. Two signals are necessary for the inflammasome to be activated. First, activation of receptors like TLRs induces the transcription of inflammasome components like Nod-like receptor, pyrin-containing (NLRP) 3, pro-IL-1β, and pro-IL-18. The second signal triggers the assembly of the inflammasome complex, consisting of NLRP3 and pro-caspase 1. This complex activates caspase 1, also known as the IL-1β converting enzyme (ICE), which leads to the maturation of IL-1β and IL-18. Caspase 1 also cleaves gasdermin-D (GSDMD) to produce the N-terminal cleavage product of GSDMD that can form transmembrane pores to release IL-1β and IL-18 into the extracellular environment and provoke pyroptosis [79].

Literature has indicated that oxidative stress promotes inflammasome assembly. For instance, treatment of LPS-primed murine bone-marrow-derived macrophages with DPI, an inhibitor of NADPH oxidase, which is responsible for producing ROS, inhibited IL-1β release caused by the oxidized phospholipid 1-palmitoyl-2-(5-oxovaleroyl)-sn-glycero-3-phosphocholine (POVPC) [80]. Moreover, the knockout of the NADPH oxidase NOX4 in human monocyte-derived macrophages decreased oxLDL-induced cell death [81].

Evidence suggests that 4-HNE inhibits the inflammasome. Nigercin is a K^+^ ionophore that activates the NLRP3 inflammasome and stimulates the death of pyroptotic cells in the presence of LPS [82]. The stimulation with nigericin and LPS induced cell death of both THP-1 and bone-marrow-derived macrophages. However, this effect was mitigated when cells were treated with 3 μM 4-HNE [83], suggesting a potential regulatory role of 4-HNE in inflammasome-driven cell death. Treatment with 3 μM 4-HNE also reduced IL-1β production in mouse peritoneal macrophages, human peripheral blood mononuclear cells (PBMC), and THP-1 macrophages in the presence of nigericin and LPS [83]. In an acute lung injury sepsis mouse model, inflammasome activation was also inhibited when treated with 6 μM 4-HNE [83].

Moreover, the inflammasome is regulated by transcription factors that are affected by 4-HNE, including Nrf2 [84] and NF-κB [36]. In THP-1 macrophages, treatment with 4-HNE led to nuclear translocation of Nrf2. However, even in the presence of the Nrf2 inhibitor ML385, 4-HNE still prevented cell death, suggesting that its effect on the inflammasome is independent of Nrf2 signaling [83]. Treatment of THP-1 macrophages with 4-HNE also did not alter p65 phosphorylation or nuclear translocation when treated with nigericin and LPS, indicating that 4-HNE modulation of the inflammasome is NF-κB independent [83].

Instead of transcriptional regulation, evidence suggests that 4-HNE might directly affect NLRP3 through protein modifications. An immunoprecipitation assay showed that pre-treatment with 3 μM 4-HNE inhibited the NLRP3 and NIMA-related kinase (NEK7) interaction in bone marrow-derived macrophages and HEK293 cells overexpressing NLRP3 and NEK7 [83]. This interaction is essential for inflammasome assembly [85]. A click chemistry-based approach was used, where cells were treated with alkyne-4-HNE, which can be conjugated to biotin by azido click chemistry. Bone-marrow-derived macrophages were treated with 10 μM alkyne-4HNE in the presence of nigericin and LPS. A pull-down revealed that alkyne-4-HNE directly bound NLRP3 in bone marrow-derived macrophages [83].

As mentioned above, caspase 1 is a member of the cysteine proteinase family that cleaves pro-IL-1β to IL-1β. Treatment of peripheral blood mononuclear cells with 4-HNE resulted in a dose-dependent decrease in caspase 1 activity, as measured by the hydrolysis of the fluorogenic substrate YVAD-AMC, strengthening that 4-HNE decreases IL-1β production [86]. Thus, 4-HNE seems to reduce inflammasome activation in human monocytes and mouse macrophages independent of Nrf2 and NF-κB. Instead, 4-HNE can directly bind to NLRP3 and prevent interaction with NEK7 [83].

### 6.2 STING signaling

The cyclic GMP-AMP synthase (cGAS)-STING pathway plays a crucial role in the innate immune response by detecting cytosolic DNA. The STING pathway is activated when two cGAS molecules recognize double-stranded DNA (dDNA), forming a dDNA-cGAs complex resulting in the conformational change of cGAS. The conformational change in cGAs enables the conversion of ATP and GTP into 2′3′-cyclic GMP-AMP (cGAMP). cGAMP binds to STING, an endoplasmic reticulum (ER) resident protein, triggering a structural rearrangement that exposes the C-terminal domain. This exposure facilitates the recruitment of TBK1, which subsequently phosphorylates IRF3. Once activated, IRF3 promotes the transcription of type I interferons (IFN–I), which exert antiproliferative and immunomodulatory effects, ultimately enhancing the immune response and restricting infection [87].

In mouse peritoneal macrophages, 6.4 μM 4-HNE induced the carbonylation of STING, which hindered its palmitoylation and prevented translocation from the ER. This disruption led to the suppression of immune responses. Glutathione peroxidase (GPX4) mediated detoxification of 4-HNE could prevent STING carbonylation, promoting STING pathway activation [88].

4-HNE not only inhibits STING, but STING singaling also promotes 4-HNE production. The impact of STING on 4-HNE production was investigated by viral knockdown of STING in mice, and their immunohistochemistry analysis revealed lower 4-HNE levels and higher GPX4 expression compared to mice without knockdown [89]. Moreover, in STING knockout mice, the levels of 4-HNE in ileal tissue were reduced upon intestinal ischemia/reperfusion injury. This shows that the STING pathway promotes 4-HNE production in mice [90].

Thus, STING promotes 4-HNE production while 4-HNE suppresses STING, suggesting a negative feedback loop. Since STING activation in macrophages triggers an inflammatory response, reduced STING activation by 4-HNE may result in a diminished inflammatory response in these cells [91].

### 6.3 COX-2

Cyclooxygenase (COX) is an enzyme that has a crucial role in the biosynthesis of prostanoids, including prostaglandins, prostacyclins, and thromboxanes, which are key mediators of inflammation, pain, and hemostasis. COX catalyzes the conversion of arachidonic acid, a polyunsaturated fatty acid, into several prostaglandins that regulate inflammation. There are two isoforms of COX: COX-1 and COX-2. COX-1 is a constitutive enzyme that maintains physiological functions such as gastric mucosal protection, platelet aggregation, and kidney function. COX-2 is an inducible enzyme primarily expressed during inflammation, contributing to pain and the inflammatory response [92,93]. Both MAPK and NF-κB signaling pathways induce COX-2 expression [94]. The canonical prostaglandin produced by COX-2 is PGE_2_, which increases the production of pro-inflammatory cytokines, including TNF-α, IL-6, and IL-1β. At the same time, PGE_2_ can also limit the pro-inflammatory response by increasing IL-10, an anti-inflammatory cytokine [95].

Stimulating RL35 epithelial cells with 25 μM of 4-HNE for 6 h increased the expression of COX-2, whereas the expression of COX-1 remained unchanged. Upregulation of COX-2 was also reported for RAW264.7 cells, although no actual data were shown in this study [96]. Another study showed elevated COX-2 expression in RAW264.7 macrophages by 4 h treatment with 4-HNE (0–75 μM). This depended on the presence of a protein in fetal bovine serum (FBS), because heat inactivation of the serum resulted in a reduction of 4-HNE-induced COX-2 expression. Oxidized low-density lipoprotein (oxLDL) was identified as a key serum component essential for 4-HNE-induced expression of COX-2. RT-PCR of RAW264.7 incubated with 4-HNE and oxLDL revealed a significant increase in scavenger receptor CD36, showing that 4-HNE induces CD36 expression, which in turn leads to increased uptake of oxLDL and elevated COX-2 expression in the presence of 4-HNE [97].

OxLDL is an inducer of atherosclerosis and foamy macrophages. Thus, it is not surprising that 4-HNE adducts and COX-2 were located in the cytoplasm of foamy macrophages in advanced atheromatous lesions of human arterial tissue. RT-PCR and immunoblotting revealed that 4-HNE could dose-dependently increase COX-2 expression, with 25 μM inducing weak expression and 100 μM 4-HNE resulting in the highest expression levels. Time-lapse experiments with 50 μM 4-HNE revealed that COX-2 expression is time-dependent and peaks between 4 and 12 h of incubation. As shown previously in RL35 epithelial cells, COX-1 expression did not change when treating RAW264.7 cells with 4-HNE [57]. Experiments with small molecule inhibitors showed that the elevated COX-2 expression was independent of NF-κB and iNOS induction but was significantly reduced when RAW264.7 macrophages were treated with Herbimycin (tyrosine kinase inhibitor), sodium orthovanadate (inhibitor of tyrosine phosphatase), PP2 (Src family tyrosine kinase inhibitor), AG1470 (EGFR like tyrosine kinase inhibitor) and SB203580 (p38 selective inhibitor) [57].

These results indicate that the accumulation of 4-HNE in murine macrophages might function as an inflammatory mediator, increasing COX-2 expression via tyrosine kinase activation, potentially through MAPK p38. Although there is no direct evidence that 4-HNE covalently binds to COX-2, its high reactivity and co-localization with COX-2 in foamy macrophages suggest that 4-HNE might potentially covalently bind to COX-2 in immune cells. Furthermore, since most of these results were obtained in murine macrophages, it would be valuable to investigate how 4-HNE affects COX-2 expression in human immune cells, especially since its effects can vary depending on the concentration and cell type.

### 6.4 Cytokine production

Given the effects of 4-HNE in many different immune signaling pathways, it is no surprise that it affects cytokine production. Exposure to 4-HNE not only suppresses inflammasome-dependent IL-1β and IL-18 production [83], as discussed above, but pre-treatment of peripheral blood monocytes with several concentrations of 4-HNE (10–50 μM) before 4 h treatment with LPS showed a dose-dependent decrease of IL-1β, IL-10, and TNF-α production. A low concentration (0–10 μM) of 4-HNE inhibited IL-1β and IL-10 production and, to a lesser extent, TNF-α, indicating that production of cytokines exhibit different sensitivities to 4-HNE [64].

However, the opposite effect of 4-HNE on cytokine production has also been reported. For example, treatment with 10 μM 4-HNE increased TGF-β1 production at both mRNA and protein levels in the human monocytic cell line U937 and the murine macrophage cell line J774-A1, while isolated Kupffer cells from cirrhotic rat liver also showed elevated TGF-β1 mRNA levels [98]. Another study shows that 4-HNE also upregulates the pro-inflammatory cytokines TNF-α, IL-1β, and IL-6 in human retinal pigment epithelial cells [99]. *In vivo*, intraperitoneal injection of 4-HNE (10 mg/kg) in wild-type C57BL/6J mice increased plasma levels of IL-6. However, this might be an indirect effect due to increased circulation of cytokine-producing immune cells, because 4-HNE treatment of wild-type mice increases circulating neutrophil counts after 4 h and circulating monocytes after 24 h [100].

Thus, the effects of 4-HNE on cytokine production are diverse. Overall, in monocytic lineage cells, 4-HNE reduces TNF-α, IL-1β, and IL-10. The suppression of TNF-α and IL-1β suggests a potential anti-inflammatory effect, further supported by the increase in TGF-β1, a cytokine known to inhibit macrophage and T cell production of these pro-inflammatory mediators. However, the reduction of IL-10, an anti-inflammatory cytokine, alongside the increase in IL-6 production, raises the possibility that 4-HNE may also exert pro-inflammatory effects. Additionally, the observed cytokine reduction could be influenced by loss of cell viability, as 4-HNE is known to induce cell death [56].

### 6.5 MCP-1

Monocyte chemoattractant protein (MCP)-1, also known as chemokine (CC-motif) ligand 2 (CCL2), plays a key role in promoting the pro-inflammatory response of macrophages, leading to inflammation. Chemokines are a family of small signaling molecules secreted by immune cells [101]. Chemokines are classified based on their chemical structure, with MCP-1 belonging to the CC family, which has cysteines closely adjoined at the N-terminus [102]. This protein attracts monocytes, which then differentiate into macrophages. These macrophages can contribute to diseases like atherosclerosis [101,103], because MCP-1 production is upregulated in response to oxLDL in human aortic endothelial cells [104].

The effects of 4-HNE on MCP-1 production are mixed. First, treatment of the J774.A1 murine macrophage cell line with several concentrations of 4-HNE (0.1–10 μM) upregulated MCP-1, with the highest increase in extracellular MCP-1 at 1 μM [105]. However, in the immortalized rat Kupffer cell line SV40, treatment with 5 μM 4-HNE did not increase MCP-1 mRNA expression. In the presence of LPS, the additional treatment with 5 μM 4-HNE resulted in a decrease in MCP-1 mRNA expression compared to LPS alone [106]. These findings highlight 4-HNE's dual role in immunity, potentially influencing macrophage-driven inflammation differently depending on the cellular context and external stimuli, although there could also be species-specific variation.

## 7 Phagocytosis and ROS production

Phagocytes are immune cells that specialize in engulfing and digesting pathogens, cellular debris, and other harmful particles. They play a crucial role in clearing infections and shaping the immune response. Primary phagocytes include macrophages, neutrophils, monocytes, and dendritic cells. These cells recognize pathogens or debris through pattern recognition receptors like TLR4 or opsonin receptors like Fc receptors and complement receptors. Once recognized, the target is enclosed in a membrane-bound vesicle, called a phagosome. The phagosome eventually fuses with the lysosome, forming the phagolysosome, where degrading enzymes and ROS, which are generated through the NADPH oxidase NOX2, can break down the engulfed material [107].

Freshly isolated human neutrophils from healthy donors were used to determine the impact of 4-HNE on phagocytosis. These neutrophils were treated with 4-HNE (0–20 μM) for 2 h before adding heat-inactivated *Staphylococcus aureus* conjugated to a fluorescent dye as phagocytic cargo. Treatment with 4-HNE led to a dose-dependent reduction of phagocytosis, with the highest decrease at 20 μM [108].

This inhibition of phagocytosis might be caused by 4-HNE protein adduct formation. Pretreatment of the RAW264.7 murine macrophage cell line with 10 μM and 20 μM 4-HNE for 1 h before incubation with pHrodo-labeled *Escherichia coli* for 3 h revealed a dose-dependent decrease in phagocytosis, with the lowest capacity at 20 μM 4-HNE. However, co-treatment with the ROS scavengers dithiothreitol (DTT) or NAC restored phagocytosis [26].

As explained above, neutrophils rapidly release ROS in response to infection, in a process called the oxidative burst, to kill engulfed pathogens [109]. Using an extracellular flux analyzer that non invasively measures cellular O_2_ consumption, it was shown that treatment of human neutrophils with 4-HNE (0–30 μM) reduced the oxidative burst dose-dependently. Using the azido click chemistry approach, where cells were first treated with 30 μM 4-HNE or 50 μM alkyne-4-HNE, followed by mass spectrometry on affinity-enriched samples, revealed the generation of multiple 4-HNE protein adducts [108]. One of the targets identified was NOX2 [108], which is involved in ROS production in the phagosome [110]. These findings suggest that 4-HNE might directly inhibit ROS production through oxidative modification of NOX2.

In summary, although there is still limited knowledge about 4-HNE's influence on phagocytosis and ROS production, these processes are inhibited in human neutrophils and RAW264.7 macrophages. Given that 4-HNE forms adducts with various proteins, this reduction is likely due to its interaction with specific proteins like NOX2. This idea is supported by the finding that phagocytosis in RAW264.7 macrophages can be rescued with antioxidants [26].

## 8 Synopsis and future directions

This review examines the multifaceted role of 4-HNE in modulating the immune response, with a focus on its impact on key signaling pathways. 4-HNE affects TLR4, MAPK, NF-κB, STING, and Nrf2 signaling in immune cells, leading to alterations in immunomodulators such as inflammasomes, cytokines, and chemokines (Table 1). These changes likely contribute to the development of inflammatory diseases, including inflammatory bowel disease [111], rheumatoid arthritis [112], atherosclerosis [17], sepsis [18], and Alzheimer's disease [16], where elevated 4-HNE levels have been observed.

**Table 1:** Distinct immunomodulatory effects of 4-HNE across immune cell types.^a^

Immune cell Type	*Cell line*	*Species*	4-HNE (μM)	*Key Effects*	*Ref*
Macrophages	RAW264.7	*Mus musculus*	0.75–20	↑↓NF-κB, ↓IRF3, ↑Nrf2,↑p38, ↓Phagocytosis,↑COX-2	[26,43,57,96,97]
	J774A.1	*Mus musculus*	0–10	↑p38, ↑TGF-β1, ↑MCP-1	[61,62,98,105]
	Bone marrow-derived	*Mus musculus*	3	↓Inflammasome	[83]
	Peritoneal	*Mus musculus*	20	↑Nrf2, ↑Prx-I, ↑p38, STING↓	[55,62,88]
	Kupffer cells	*Rat norvegicus*	10	↓NF-κB, ↓IL-6, ↑TGF-β1, ↓MCP-1	[45,98,106]
	Foamy	*Homo sapiens*	25–100	↑COX-2	[57]
	Alveolar	*Mus musculus*	25–50	↑Nrf2	[56]
Monocytes	THP-1	*Homo sapiens*	5–50	↓NF-κB, ↑p38, ↓Inflammasome	[44,63,83]
	U937	*Homo sapiens*	5–50	↑TLR4, ↑IL-1β, ↑IL-8, ↑TNF-α, ↑TGF-β1	[29,64,83]
	MonoMac6	*Homo sapiens*	10	↓p38, ↑JNK, ↓ERK	[64]
	Peripheral blood	*Homo sapiens*	10–50	↓p38,↓NF-κB, IL-18↓, IL-1β↓, IL-10↓, TNF-α ↓	[44,64]
T cells	Jurkat	*Homo sapiens*	30	↓NF-κB	[46]
	CRL2571	*Homo sapiens*	5, 20	↑JNK	[68]
Neutrophils	Peripheral blood	*Homo sapiens*	0–20	↓Phagocytosis	[108]
B cell	Ba/F3	*Mus musculus*	10, 20	↑TLR4	[26]

a ↑ = increase, ↓ = decrease; Ref = reference.

For instance, in atherosclerosis, MCP-1 and TNF-α are two inflammatory mediators released during foam cell formation [113]. As 4-HNE is known to affect TNF-α and MCP-1 production and is increased in atherosclerotic lesions, 4-HNE might well contribute to foam cell formation. The upregulation of TGF-β by 4-HNE could also indicate that 4-HNE contributes towards the severity of fibrotic diseases, as TGF-β is increased in fibrotic diseases [114]. Moreover, 4-HNE can modify amyloid-β peptides, preventing their proteasomal degradation, which may play a significant role in the progression of Alzheimer's disease [115].

However, the effect and contribution of 4-HNE on the immune response likely vary among pathologies, as its effect varies based on cell type and concentration. As discussed in this review, 4-HNE exhibits complex regulatory effects on immune signaling, including up- and down-regulation of TLR4 signaling, NF-κB activation at low concentrations and suppression at high concentrations, and upregulation of Nrf2 by inhibiting KEAP1. It also differentially influences MAPK pathways, promoting p38 and JNK activation while downregulating ERK. Additionally, 4-HNE downregulates the inflammasome and STING signaling, enhances COX-2 expression, and modulates cytokine levels while suppressing phagocytosis and ROS production. It is important to understand the dose- and cell type-dependent effects of 4-HNE on the development of diseases. A dose-response curve for different immune cells will shed light on the precise effects of 4-HNE and is required for a full understanding of how it contributes to inflammatory processes. Furthermore, these findings should be compared to *in vivo* for confirming the responses of different cell types to 4-HNE, and also for revealing potential cross-talk between immune cells and other tissue cells.

The differences in the effects of 4-HNE on different immune cell types could be explained through differences in the antioxidant system, identical pathways driving divergent functional effects, or differences in the use of immortalized cell lines versus the use of primary cells. For example, 4-HNE induced a dose-dependent increase in NF-κB activity in RAW264.7 cells [43], whereas in primary rat Kupffer cells, it led to a dose-dependent suppression [45]. This discrepancy could reflect intrinsic differences in redox metabolism between immortalized and tissue-resident macrophages. RAW264.7 cells are derived from cancer cells and carry mutations that allow continuous division [127], making them fundamentally different from tissue-resident cells like Kupffer cells. Cancer cells typically produce higher levels of ROS to support their growth and survival, and as a result, they develop strong antioxidant defenses [128,129]. This may make RAW264.7 cells less sensitive to oxidative stress compared to primary macrophages. This underscores the importance of cell context in interpreting redox-sensitive signaling pathways. It also shows that different cells can act differently towards distinct ligands due to the activation of distinct signaling molecules, a notion best illustrated by the finding in THP-1 cells that co-treatment with 4-HNE and LPS, PMA, or TNF-α resulted in different outcomes [44]. Therefore, the role of 4-HNE in signaling pathways across immune cell types should not be generalized, as signaling networks are highly cell-type-specific and involve complex, interconnected pathways. These networks should be characterized individually in each immune cell type, preferably using primary human cells, for more accurate and translatable insights into human physiology.

Given the increasing concentrations of 4-HNE in oxidative stress-related diseases, targeting 4-HNE production could be a promising therapeutic strategy. Antioxidants could neutralize 4-HNE, while enhancing detoxifying enzymes like aldehyde dehydrogenases and glutathione S-transferase may reduce its harmful effects. Interestingly, a monoclonal antibody against 4-HNE has demonstrated protective effects in mice during liver injury and endotoxemia, highlighting its potential as a therapeutic target for various diseases [116].

However, given the diverse effects of 4-HNE on immune responses, increasing 4-HNE levels could also be a therapeutic strategy. In a mouse model for sepsis, the oropharyngeal administration of 6 μM 4-HNE in an acute lung injury mouse model demonstrated beneficial effects through the inhibition of the inflammasome [83]. In patients with sepsis, lipid peroxidation products like 4-HNE and MDA are increased [18]. However, their roles in the development and survival rate of the disease remain unclear.

While most research has focused on the short-term effects of 4-HNE, its long-term impact on chronic diseases remains largely underexplored, despite the potential for cumulative cytotoxicity due to the accumulation of 4-HNE-protein adducts and increased ROS production. Investigating prolonged exposure to 4-HNE is crucial, especially in different immune cell subsets, to understand 4-HNE's contribution to age-related pathologies.

Moreover, the effects of 4-HNE on immune signaling likely not only affect cytokine production, ROS production, and phagocytosis, as discussed in this review, but also other cellular functions. It is known that both extracellular and intracellular ROS modulate individual immune cell metabolism, signaling, and survival, which are regulated by cellular antioxidants like glutathione, thioredoxin, HIF-1α, and NRF-2 [117]. For example, since NF-κB and p38 MAPK are critical for neutrophil survival, interference by 4-HNE with these pathways may induce apoptosis [118]. Together with the direct inhibition of phagocytosis by 4-HNE [108] this will lower neutrophil functions.

Two complications are that 4-HNE is never the sole product produced by lipid oxidation, and that lipid oxidation also changes the membrane composition. For example, in RAW264.7 murine macrophages, 4-HNE increases ROS production, which react with arachidonic acid, an essential lipid for membrane remodeling and pseudopod extension [119], potentially impairing not only phagocytosis but also cell migration. Moreover, in airway and alveolar macrophages, the oxidation of glutathione impairs phagocytosis and prevents cell survival [120,121], which might contribute to the decrease in phagocytic activity seen in RAW264.7 [26].

The finding that the effects of 4-HNE differ between immune cell types is in line with the different effects of antioxidants on immune cell types and their different sensitivities towards ROS [117]. For example, elevated levels of microenvironmental ROS promote Th2 polarization of helper T cells [122], whereas low ROS levels favor the differentiation of Th1 and Th17 cells [123]. While hydrogen peroxide is necessary for proper B-cell receptor maturation [124], excessive ROS can disrupt redox balance and impair B-cell functionality [125]. As the increase in ROS production is linked to an increase in 4-HNE [126], it is expected that 4-HNE affects distinct immune cells differently due to cell-intrinsic redox capacity and survival mechanisms. Thus, future research should address the effects of 4-HNE on different signaling pathways, with their effect on immune cell functions like cell migration, cell survival, and antigen presentation.

In conclusion, 4-HNE exerts diverse immunomodulatory effects by influencing key signaling pathways, including TLR4, NF-κB, MAPKs, STING, and Nrf2. Its impact varies depending on concentration and cell type, making it a highly dynamic regulator of immune responses and inflammation. Further research into these pathways and the interactions between different immune cell types will be essential for developing therapeutic approaches to target 4-HNE in inflammatory diseases.

## CRediT authorship contribution statement

**Melina Ioannidis:** Writing – original draft. **Johanna Tjepkema:** Conceptualization. **Michael R.P. Uitbeijerse:** Conceptualization. **Geert van den Bogaart:** Funding acquisition, Resources, Writing – review & editing.

## Funding

GvdB has received funding from the European Research Council (ERC) under the European Union's Horizon 2020 research and innovation program (grant agreement No. 862137), and ZonMW (project grant No. 09120011910001).

## Declaration of Competing interest

The authors declare no conflict of interest.

## Data Availability

Data will be made available on request.
